# Demonstration of cross reaction in hybrid graphene oxide/tantalum dioxide guided mode resonance sensor for selective volatile organic compound

**DOI:** 10.1038/s41598-023-37795-6

**Published:** 2023-07-04

**Authors:** Khwanchai Tantiwanichapan, Romuald Jolivot, Apichai Jomphoak, Nantarat Srisuai, Chanunthorn Chananonnawathorn, Tossaporn Lertvanithpol, Mati Horprathum, Sakoolkan Boonruang

**Affiliations:** 1 Spectroscopic and Sensing Devices Research Group (SSDRG), Opto-Electrochemical Sensing Research Team (OEC), National Electronics and Computer Technology Center (NECTEC), Pathum Thani, 12120 Thailand; 2 School of Engineering, BU-CROCCS, Bangkok University, Pathum Thani, 12120 Thailand

**Keywords:** Materials science, Optics and photonics

## Abstract

This paper experimentally demonstrates a crossed reaction of pure and hybrid graphene oxide (GO)/tantalum dioxide (TaO_2_) as a volatile organic compound (VOC) absorber in a guided mode resonance (GMR) sensing platform. The proposed GMR platform has a porous TaO_2_ film as the main guiding layer, allowing for more molecular adsorption and enhanced sensitivity. GO is applied on top as an additional VOC absorber to increase the selectivity. The hybrid sensing mechanism is introduced by varying the concentration of the GO aqueous solution. The experimental results show that the pure TaO_2_-GMR has a high tendency to adsorb most of the tested VOC molecules, with the resonance wavelength shifting accordingly to the physical properties of the VOCs (molecular weight, vapor pressure, etc). The largest signal appears in the large molecule such as toluene, and its sensitivity is gradually reduced in the hybrid sensors. At the optimum GO concentration of 3 mg/mL, the hybrid GO/TaO_2_ -GMR is more sensitive to methanol, while the pure GO sensor coated with GO at 5 mg/mL is highly selective to ammonia. The sensing mechanisms are verified using the Density Functional Theory (DFT) to simulate the molecular absorption, along with the measured functional groups measured on the sensor surface by the Fourier transform infrared spectroscopy (FTIR). The crossed reaction of these sensors is further analyzed by means of machine learning, specifically the principal component analysis (PCA) method and decision tree algorithm. The results show that this sensor is a promising candidate for quantitative and qualitative VOCs detection in sensor array platform.

## Introduction

For the past few decades, the demand on monitoring of volatile organic compounds (VOC) has dramatically increased and has applied in many fields. VOC originate from both natural (plants, bacteria, etc.) and anthropogenic sources (fuel production, fuel combustion, general industries, cosmetics, personal care-products)^1^. Most VOC have high toxicities and are harmful to human health with effects ranging from headache, eye, throat, and nose irritation for short time exposure to long term chronical disease^2,3^. Consequently, many countries have their own pollution-controlled regulations for each VOCs level and measurement^4,5^. On the other hand, monitoring of VOC can benefit the food industry for quality control and assessment of food, beverage, and cosmetics^6^. A study in the medical field has also associated the detection of specific VOC emission via breath or skin with different diseases such cancers, rheumatoid arthritis, etc^7^.

As of today, the gold standard for VOC detection uses gas chromatography coupled with mass spectrometry (GC–MS)^8^. This technique provides highly sensitive and accurate analytical measurements of VOC delivering the detection, identification, and quantification of individual VOC or even mixtures of VOC. While being highly efficient, GC–MS is a time-consuming and expensive method that requires skilled technicians to operate it. Hence, on-going research has aimed to develop low-cost, fast, reliable, and sensitive sensors for the detection and quantification of VOC^9^. Many techniques have been investigated such as gravimetric^10^, chemoreceptive^11^, and optical sensor^12–17^, etc. Nevertheless, selectivity remains a challenge to identify the VOC especially in a mixed VOC scenario.

Electronic or optoelectronic nose is one promising platform to recognize the VOC by mimicking mammal’s olfactory mechanisms^20–24^. That requires a cross-reactive sensor array configuration for obtaining the VOC fingerprint and a high throughput data analysis for decision making. For this purpose, various sensing materials (metal oxide, 2D material, conductive polymer, and carbon, etc.) have been intensively explored to distinguish the signal for the specific VOC^25,26^. It is worth noting here that increasing the number of sensor arrays can contribute to more complex signal recognition and therefore improve accuracy in data interpretation. Among the referred sensing techniques, the optical platform has more advantage as the sensor geometry is less complex and it is easy to fabricate. In the literature, optoelectronic noses based on colorimetric and fluorescence sensor array^12^ have been intensively and practically demonstrated in many applications. However, such sensor is not reusable, and it has low stability. In order to overcome this fact, a variety of label-free sensing techniques have been demonstrated, such as fiber optic waveguide array^22^, surface plasmon resonance (SPR) imaging^23^, and on-chip waveguide-array interferometer^24^. Another promising technique applies guided mode resonance (GMR) concept in both optical fiber and on-chip configuration^15–19^.

In the GMR sensor, the device configuration comprises of a subwavelength grating embedded in a waveguide structure. Resonance occurs when the phase matching condition between diffracted waves and guided mode is met. That introduces strong reflection with spectral and angular selectivity. Resonance relocates proportionally to the structure’s optical properties and dimensions. Hence, molecular adsorption on the surface can be measured through the shift of resonance signal using spectrum, angular or intensity interrogation method. GMR has been constantly demonstrated especially in biomolecular sensing applications^27^. There exist however few works in VOC/gas detection^15–19^. Recently, Tabassum et al. proposed a GMR fiber sensor made of titanium dioxide (TiO2) for VOC/gas detection. The sensor was coated with gas adsorption material such as graphene oxide (GO) nanosheets for demonstration of gaseous ethylene and methanol^15^ detection and copper (I) complex for monitoring ethylene-promoted ripening in banana^16^. In another experimental work, Lin et al. has integrated the palladium (Pd) film on a planar TiO_2_ waveguide-GMR for hydrogen gas sensing^17^. The sensitivity of the GMR sensors can be improved by increasing light-matter interaction in alternative designs, such as by inserting VOCs sensing cavity inside the GMR structure^18^ or by creating void areas in the main guiding film^19^.

As aforementioned, metal oxide semiconductors, such as tungsten oxide (WO_3_), tin oxide (SnO), zinc oxide (ZnO), etc., are well established for gas/VOC sensing. Due to their excellent optical properties, they are also applied in many of the optical devices. Lately, nanocolumnar tantalum oxide waveguide^19^ has been introduced in GMR sensor for VOC detection with significant enhancement of sensitivity. The sensor however has low selectivity. This constraint can be further improved by means of cross reaction in a sensor array form. Additional VOC/gas sensing materials are required to generate variance in the signal. For this purpose, this paper includes a study of the cross reaction of hybrid TaO_2_/GO -GMR sensors. By varying GO concentrations, the TaO_2_ waveguide-GMR sensors are coated with different percentage coverage generating a unique signal pattern of each VOCs that can be further classified by means of machine learning.

The sensors in this study were fabricated and tested with different groups of VOCs (isopropanol, ethanol, methanol, acetone, toluene, ammonia) using the interrogation of resonance wavelength shift. The fabrication method, materials, and measurement set up are described in detail in Section “Fabrication and experiment”. Section “Result and discussion” includes the experimental results, as well as the surface and material characterizations. The sensing mechanisms are clarified using the Density Functional Theory (DFT) to simulate the molecular absorption along with the measured functional groups on the sensor surface obtained through Fourier transform infrared spectroscopy (FTIR). Principal component analysis (PCA) was used to confirm distinguish able data classification, supporting the use of the proposed hybrid sensors in an array platform for optoelectronic nose.

## Fabrication and experiment

### Hybrid GO/TaO_2_ GMR fabrication

**Figure 1:**
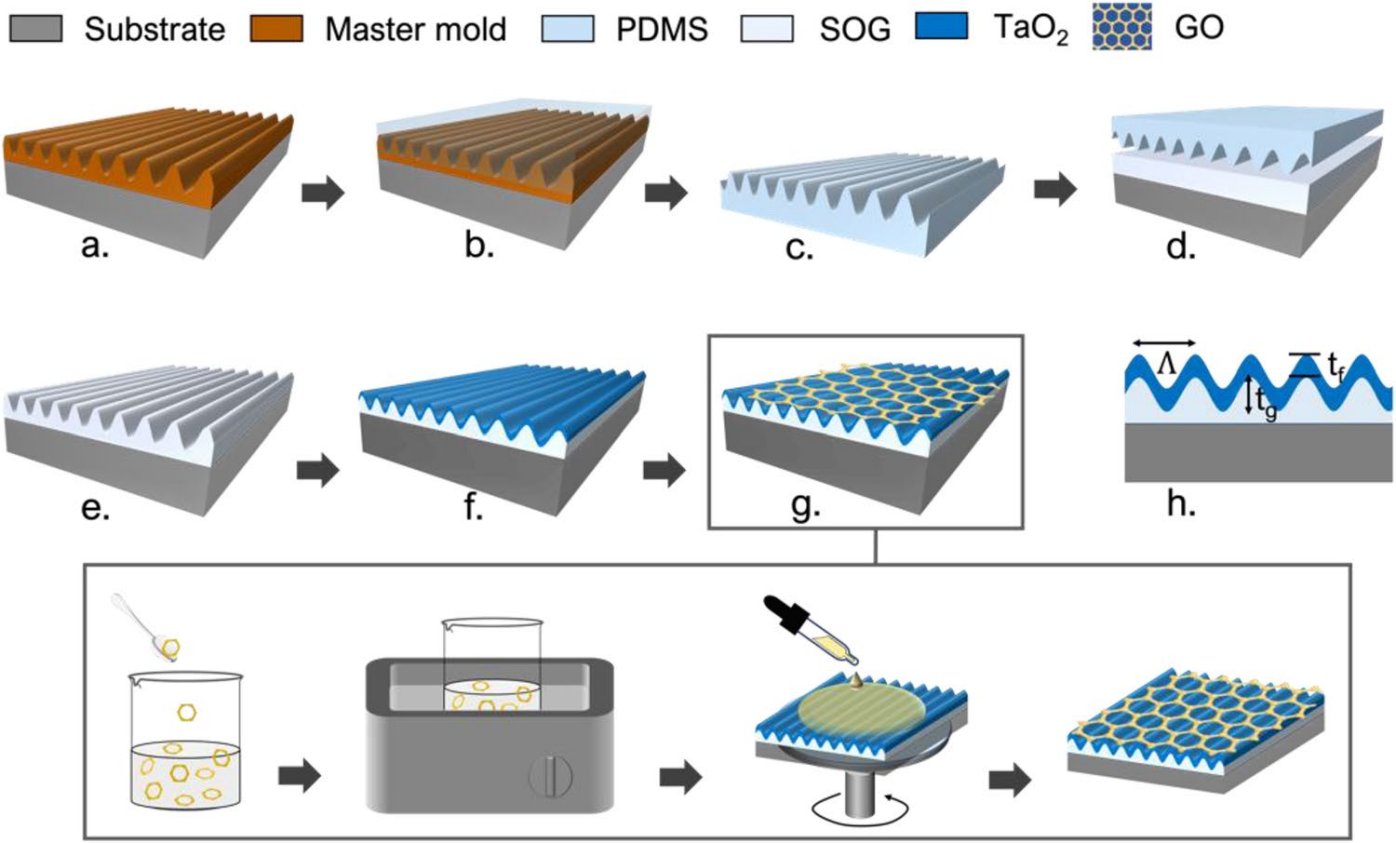
Schematics diagram of the hybrid GO/TaO_2_-GMR fabrication process.

The fabrication of the GMR-based VOC sensor involves a multilayer grating-waveguide structure on a transparent substrate. As illustrated in Fig. 1, the process starts from a formation of the subwavelength grating structure on a low refractive index film using a thermal-cured nanoimprint method. The imprinted grating is then coated with a refractive index film to form a waveguide layer using a sputtering technique. As illustrated from Fig. 1a,b, the nanoimprint process requires the preparation of an imprinting mold with a casting method using a polydimethylsiloxane (PDMS) elastomer (Sylgard 184 silicone elastomer from Dow corning). The one dimensional (1D) grating master mold (Fig. 1a) with sinusoidal profile has previously fabricated by laser interference lithography (LIL). The PDMS grating replica, shown in Fig. 1c, is subsequently used as the main mold for further fabrication of the GMR sensors. At the same time, the thermal-cured nanoimprint resist (spin-on-glass, SOG (400F, Filmtronics Inc.) is then coated on a glass slide substrate by spin coating method, and the grating structure from the PDMS mold is imprinted on the SOG film as shown in Fig. 1d and e. A TaO_2_ film is deposited on top of it using a commercial pulsed DC magnetron sputtering technique (AJA International, Inc. ATC 2000-F). In the sputtering process, tantalum (Ta:99.995%) target is sputtered by argon (Ar) atoms under the operating pressure of 20 mTorr and oxygen (O2) gas is supplied in the chamber to create the nanocolumnar tantalum oxide film^19^. The detailed methods and parameters are described in the supporting information S1. The GMR sensor is constructed with geometry illustrated in Fig. 1h.

The introduction of graphene oxide (GO) on top of the TaO_2_-GMR surface creates the hybrid sensors as shown in Fig. 1g. GO is coated on the TaO_2_-GMR sensor’s surface in different concentrations, ranging from 1 to 5 mg/mL. Highly concentrated graphene oxide (5 mg/mL and the flake’s size around 0.5–5 μm) purchased from Graphene Supermarket (part no: SKU-HCGO_W_60) is diluted with deionized (DI) water. The dilution for each of the five concentration is performed by sonicating for 90 min to ensure the homogenous distribution of GO flakes in a solution. Then, 400 μL of each GO concentration is coated on each TaO_2_-GMR sensor’s surface using a spin coater. The surface of the sensor is first activated with an oxygen (O_2_) plasma to enhance the adhesion before spin coating GO at 2000 rpm for 60 s. All GO-coated GMR sensors are slowly dried out at ambient environment for 24 h. As a result, different concentrations of GO coated on TaO_2_-GMR sensors are fabricated.

The deposited films are later characterized using X-ray photoelectron spectroscopy (XPS) and Raman spectroscopy (Renishaw InVia Reflex) to confirm the material and molecular structure, while the physical morphology is measured using field emission scanning electron microscopy (FE-SEM: HITACHI SU8030).

### VOC sensing setup

**Figure 2:**
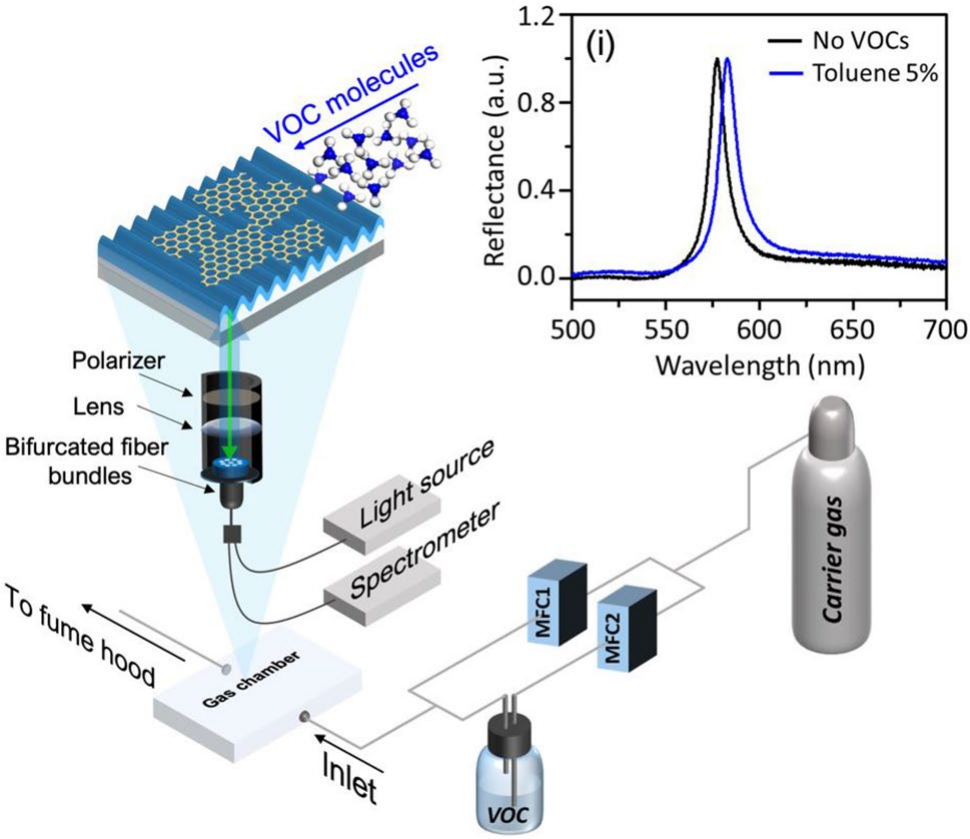
VOC -test optical setup showing the VOC feeding in the sensor chamber. (i) Reflection spectrum without VOC and with 5% toluene exposure.

The sensitivity of the GMR sensor to VOC is measured experimentally using a resonance spectroscopy and a vapor-phase VOC generator/controller as shown in Fig. 2. The optical setup in this study uses a broadband tungsten-halogen light source (Ocean Optics: HL2000) coupled into bifurcated fiber bundles. Light beam is first configured using a collimating lens and a linear polarizer before being normally incident onto the substrate side of the GMR sensor. To ensure high sensitivity. The polarizer is set to excite only TM resonance mode. The reflected light is coupled back to another core of the fiber probe for real-time detection of the resonance reflection spectrum using a spectrometer (Thorlabs: CCS200). The resonance signals are later extracted and analyzed with the method described in Section "Signal acquisition". The GMR sensor is encapsulated in a tested-VOC vapor flow chamber, where the vapor is formed using a bubbling technique. Two separated mass flow controls (MFC1 and MFC2) are used to regulate the flow rate of the carrier gas (air zero containing 21% oxygen balance Nitrogen and no water vapor) purging into the chamber directly and into the VOC solution, respectively. The VOC concentration and sample feeding protocol can be automatically controlled through the customized MFC graphical user interface software.

To measure the selectivity of each sensor, six type of VOCs (isopropanol, ethanol, methanol, ammonia, acetone, and toluene) are tested at a fixed concentration of VOC for four measurement loops. Each measurement loop consists of an exposure to 100% of carrier gas for a period of 5 min followed by an exposure to 5% of VOC for another period of 5 min. The resonance spectra obtained from the referred spectroscopy is recorded every 3 s during each measurement.

### Signal acquisition

The resonance reflection spectra acquired at each time stamp (t_i_) using Thorlabs OSA software are processed following the algorithm described in the supporting information S2. First, noise reduction is performed using a moving average algorithm where the reflectivity at each wavelength is set to the average of the values from a window of N_s_ points around it. The smoothed data is then cropped according to a manually selected range of detection with the window of width N_f_ around the maximum intensity. The peak of the fitted data obtained using second order polynomial fitting of the signal indicates the resonance wavelength (${\lambda }_{0}$). The kinetic responses of the sensor during VOC measurements are measured by extracting four parameters (${\Delta \lambda }_{01}$, ${\Delta \lambda }_{02}$, ${\Delta \lambda }_{03}$, ${\Delta \lambda }_{0c}({t}_{i})$) as shown in Fig. S2ii from the resonance time plot. The processing steps includes noise reduction from the resonance time plot (${\lambda }_{0}(t)$), baseline subtraction to convert the filtered resonance time plot (${\lambda }_{0}(t))$ into the resonance shift ${(\Delta \lambda }_{0}(t))$, and auto-detection of desired signal for further computation.

Here, four key points are automatically detected. ${\Delta \lambda }_{01}$ corresponds to the signal at the starting point when VOC is injected into the chamber at t_1_, ${\Delta \lambda }_{03}$ refers to the highest signal recorded at t_3_ before releasing VOC from the chamber, ${\Delta \lambda }_{02}$ having value of 90% of ${\Delta \lambda }_{03}$ represent the point when the signal starts to reach saturation at t_2_, and ${\Delta \lambda }_{0c}({t}_{i})$ is the signal at each t_i_ while VOC is fed in the chamber. Using the four detected points, three metrics (${\Delta \lambda }_{100\%}$, ${\Delta \lambda }_{90\%}$, S_c_) are then calculated as defined in Eqs. (1), (2), and (3), respectively.

1 $${\Delta \lambda }_{100\%}={\Delta \lambda }_{03}-{\Delta \lambda }_{01}.$$

2 $${\Delta \lambda }_{90\%}={\Delta \lambda }_{02}-{\Delta \lambda }_{01}.$$

S_c_ represents to the kinetic response of the sensor while it is exposed to the VOC. The calculation follows the Eq. (3), where it takes N-point data from t_2_ to t_3_ to estimate the slope of the signal.

3 $${S}_{c}\,=\,\frac{1}{N}\sum_{i=1}^{N }{S}_{ci}\, and\, {S}_{ci}\,=\,\frac{{\Delta \lambda }_{0c}({t}_{i})-{\Delta \lambda }_{0c}({t}_{i-1})}{{t}_{i}-{t}_{i-1}},\, where\, i\,=\,\mathrm{1,\,2\,},\ldots , \,N$$

## Result and discussion

### Device and material characterization

**Figure 3:**
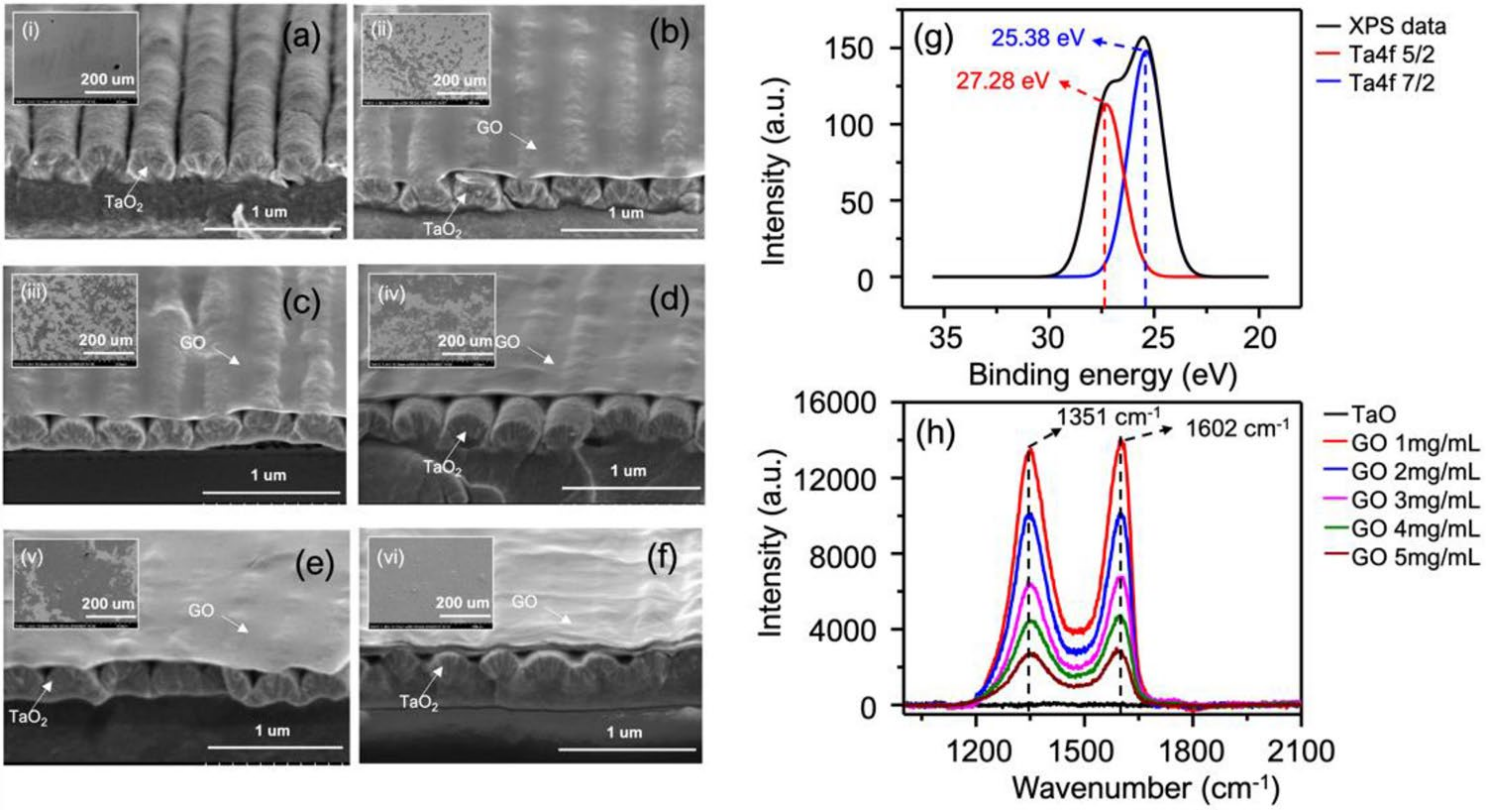
FE-SEM images (top and bird-eye view) of each sensor (**a**) pure TaO_2_ (**b–f**) GO 1, 2, 3, 4, 5 mg/mL coated on TaO_2_—GMR (**g**) XPS measurement of TaO_2_ film coated with operation pressure of 20 mTorr (**h**) Raman spectrum of each sensor.

The FE-SEM images in Fig. 3a–f demonstrates the morphology of the six fabricated TaO_2_-GMR sensors, with and without GO coating at varying concentrations from 1 to 5 mg/mL. The detailed layer structure and dimensions are included in the supporting information S3. As shown, the based TaO_2_-GMR sensor is constructed with a SOG sinusoidal surface grating having a measured period of around 360 nm and a measured depth of around 100 nm, where the unpatterned SOG film under the grating has a thickness of around 350 nm. At high operating pressure deposition, the TaO_2_ film appears to have a nanocolumnar structure with a measured thickness of around 190 nm. As presented in the previous work^19^, the optical properties and morphology of the film at this condition has a refractive index of 1.996 and a pore size of 4.47 ± 1.1 nm, which represents 14.43% porosity. This allows more VOC molecules to penetrate inside, enhancing the sensitivity. Furthermore, the XPS measurement in Fig. 3g confirms the formation of TaO_2_ film having a binding energy at 27.28 and 25.38 eV.

**Figure 4:**
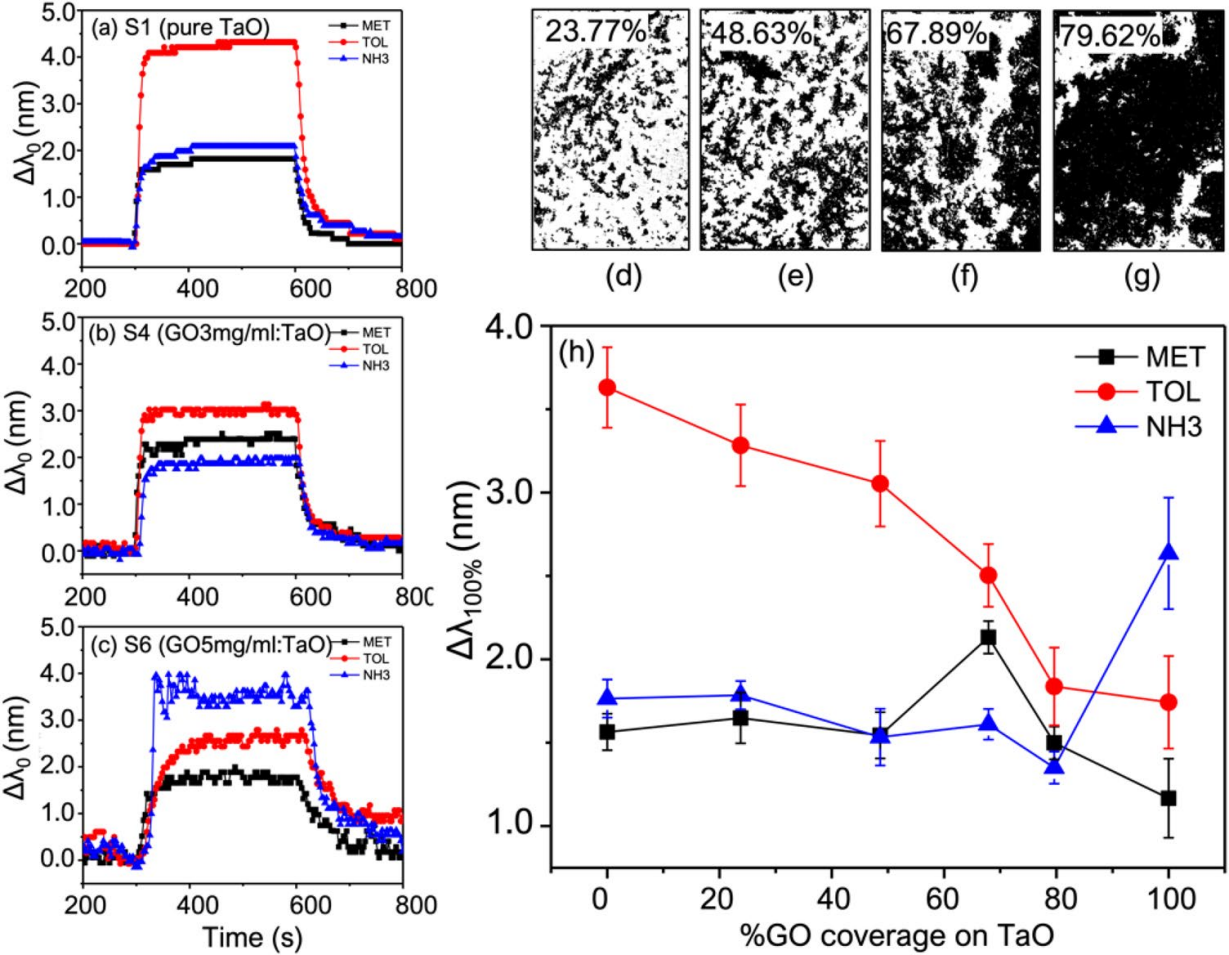
Compared time plots of the resonance shift in single-loop measurement of methanol, toluene, and ammonia using the sensor with (**a**) pure TaO_2_ (**b**) GO 3 mg/mL coated on TaO_2_ (**c**) GO 5 mg/mL coated on TaO_2_. The processed SEM images to show GO coverage of (**d**) 23.77% (**e**) 48.63% (**f**) 677.89% and (**g**) 76.62% on the sensors with GO coating of 1, 2, 3, and 4, respectively. (**h**) The shift of the resonance versus percentage of GO coverage when testing with methanol, toluene, and ammonia.

FE-SEM measurements were used to demonstratively confirm the coverage area and thickness of GO coated on the TaO_2_-GMR for the hybrid sensors with varying concentrations of GO ranging from 1 to 5 mg/mL. Raman spectroscopy was employed to ensure the existence of GO. As shown in the inset (ii) to (vi), the results revealed a non-uniform deposition of GO. At low concentration (GO < 4 mg/mL), some void areas without GO deposition were observed, while the other areas are aggregated with GO. This essentially allows the VOC molecular interaction with both TaO_2_ and GO. This hybrid sensing mechanism is allowed for enhanced signal recognition in the sensor array platform. The percentage of GO coverage however was estimated using image processing methods. Within the calculated area of 500 × 500 μm as shown in the processed SEM images in Fig. 4d–g. The calculations based on image processing indicate that the coverage of GO increases from 23.77%, 48.68%, 67.89%, and 79.62% on the sensors coated with an increase of GO concentration at 1, 2, 3, and 4 mg/mL, respectively. The sensor coated with 5 mg/mL GO has full coverage with a film thickness of 48 nm as presented in Fig.S3. The Raman spectrum in the plot Fig. 3h shows the two Raman peaks of GO located at G (1602 cm^−1^) and D (1351 cm^−1^) band. The signal intensity in the Raman spectrum decreases with an increase in the number of GO layers on the sensors^28^.

It is worth noting here that although GO has small absorption (k < 0.1^29^) in the visible spectrum, deposition of GO on the TaO_2_-GMR results in a red-shift of the resonance spectrum as well as decrease in the resonance intensity especially in the sensor coated with high concentration of GO. The compared resonance spectra of the fabricated sensors are included in the supporting information S4. The signal processing approach presented in Section "Signal acquisition" is hence necessary to extract the signal accurately for further analysis.

### VOC optical measurement

As mentioned in the Section "VOC sensing setup", the six sensors are individually tested with six different types of VOC at fixed concentration of 5% and four measurement cycles. The time plots of all measurements are included in the supporting information S5. Here, only relevant measurements are demonstrated in the plots Fig. 4 to verify the sensing ability in the pure and hybrid sensors. The resonance time plots in Fig. 4a–c demonstrate the change of the resonance wavelength in a single-loop measurement of three VOCs (methanol, toluene, and ammonia) in comparison for the pure TaO_2_ sensor without GO coated, the hybrid sensor with 3 mg/mL GO coated, and the pure GO sensor with full coverage of GO, respectively.

As demonstrated, the pure TaO_2_ sensor is highly sensitive to the toluene, with a large resonance wavelength shifted up to 4 nm is measured. Meanwhile, the sensor is less sensitive to methanol and ammonia, with a similar signal level. On the other hand, the hybrid sensor with 67.89% GO coverage (3 mg/mL) appears to detect methanol more efficiently but it is less sensitive to toluene. For the pure GO sensor, the ammonia signal is dramatically enhanced, while the toluene signal continues to decrease. Following the signal acquisition method in Section "Signal acquisition", the resonance shifts (${\Delta \lambda }_{100\%}$) are estimated and plotted against the percentage of GO coverage in each sensor, as shown in Fig. 4h. The results confirm the reduction of toluene signal in the presence of more GO coverage. In this regard, toluene seems to have better molecular adsorption on the TaO_2_ surface. The applied GO has a high tendency to prevent molecular penetration into the waveguide surface. On the other hand, GO is highly sensitive to ammonia. The hybrid sensor, however, does not show an increase in the ammonia signal, but rather an increase in the methanol signal at the optimum concentration.

The sensitivity of these sensors when tested with the three VOCs is determined with a similar feeding sequence but varying the vapor concentrations (2.5, 5, 7.5, 10, 15 and 20%). The resonance wavelength shifts proportional to the VOC concentrations as depicted in Fig. S6a. As shown, at concentrations higher than 10%, the graphs become nonlinear. The sensitivity and the limit of detection (LOD) are then calculated using the data in the linear range for concentrations less than 10%. The calculated results are plotted in Fig. S6b–d. It is shown that pure TaO_2_ sensor (S1), hybrid sensor (S4), and pure GO sensor (S6) are the most sensitive to toluene, methanol, and ammonia with LOD of 0.089%, 0.0192%, and 0.0297%, respectively.

**Figure 5:**
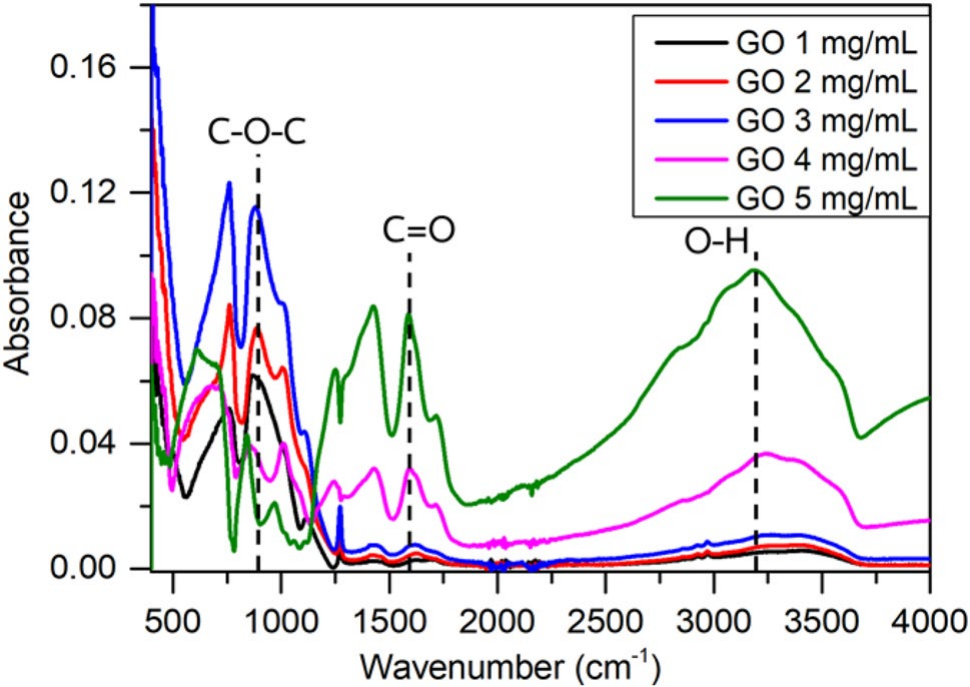
FT-IR (ATR mode) measurement for different GO concentration showing functional groups appeared on the fabricated GMR sensors.

Theoretically, the resonance in the GMR sensor shifts proportionally to the change in optical properties of the film, caused by molecular adsorption and variations in the environment. The number of the adsorbed molecules and their physical properties (molecular weight, vapor pressure, and refractive index, etc.) contribute to the sensitivity of the sensor. Additional binding sites are necessary to enhance the selectivity. In the pure TaO_2_-GMR, the nanocolumnar TaO_2_ film allows more VOC to diffuse inside the waveguide film introducing more light-matter interaction and hence increase in sensitivity. However, there does not exist dominant functional group on the TaO_2_ film as presented in FT-IR measurement in Fig. 5. Thus, the physical absorption becomes more dominant. The experimental results show detectable signals with the magnitude corresponding to the physical properties of VOCs (see the supporting information S7).

Meanwhile, GO is well known as an excellent candidate for VOC/gas sensors due to its intrinsic functional groups^30^. These functional groups enhance molecular interactions, leading to increased adsorption during VOC/ gas exposure. To verify the functional groups of the GO deposited at each concentration, the sensors are characterized by ATR measurement in FT-IR spectroscopy as plotted in Fig. 5. The result shows a strong signal of hydroxyl (O–H) and carboxyl (C = O) group observed from the absorbance located at 3200–3400 cm^−1^ and 1600–1750 cm^−1^, respectively. As shown, the absorbance value at each band increases proportionally to the GO concentration. Another dominant signal representing the epoxy (C–O–C) group at 750–950 cm^−1^ is strengthened with respect to the concentration and maximized at 3 mg/ml. However, the epoxy group becomes diminished at higher concentration of 4 and 5 mg/ml.

**Figure 6:**
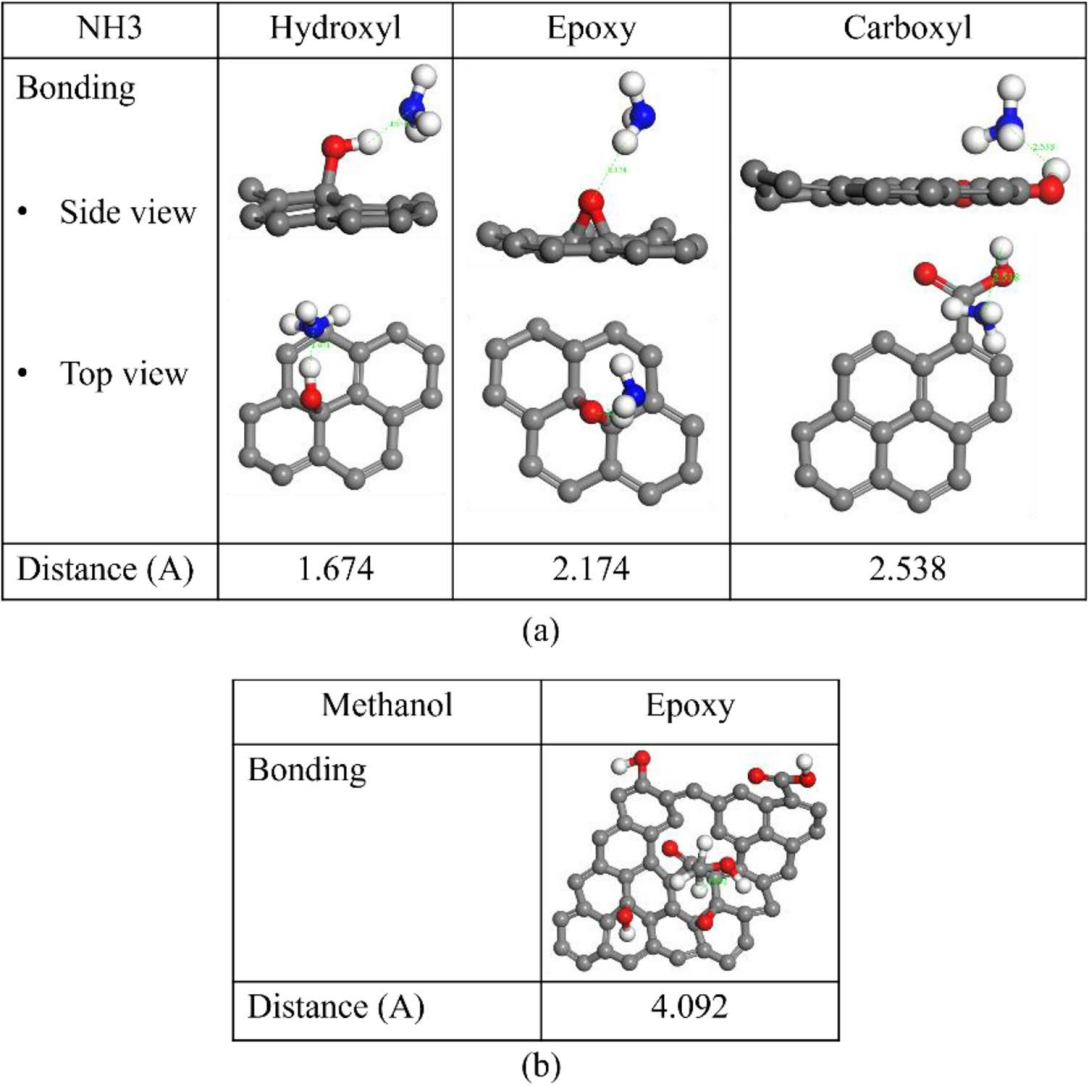
DFT simulation results of (**a**) NH_3_ adsorption on the GO with hydroxyl, epoxy and carboxyl group (**b**) methanol adsorption on the GO with epoxy group.

To clarify this, Density Functional Theory (DFT) is used to further analyze the VOCs molecular adsorption distance on GO with hydroxyl, epoxy and carboxyl groups. In this work, the generalized gradient approximation (GGA) is used to mainly describe the van der Waals interaction^31^. To simplify the computation, the interaction of each functional group and only relevant VOC molecules (ammonia and methanol) based on the measurement is simulated individually. According to Fig. 6, NH_3_ is attached to the hydroxyl, epoxy, and carboxyl in GO with distances of 1.674 A, 2.174, and 2.538 A, respectively. The DFT indicates that N atom of NH_3_ easily adsorbs to H atom in hydroxyl group in GO. The results from FT-IR and DFT show similar trend with the experimental results. The sensor with GO concentration of 5 mg/mL covered on TaO_2_ exhibited the highest NH_3_ sensitivity, attributed to the presence of hydroxyl functional groups in GO, as shown in Fig. 4. Additionally, methanol molecule absorption is observed through the same method. The methanol molecule demonstrates a preference for epoxy group, specifically towards the oxygen atom of epoxy group in GO, with a minimum distance of 4.092 A. This DFT result is consistent with the FTIR function group analysis and experimental findings. The sensor with a GO concentration of 3 mg/mL exhibits the highest epoxy functional group and maximum methanol sensitivity. In contrast, sensors with the larger GO concentrations (i.e. 4 and 5 mg/mL.) that have fewer epoxy group show lower methanol sensitivity.

### Data classification

**Figure 7:**
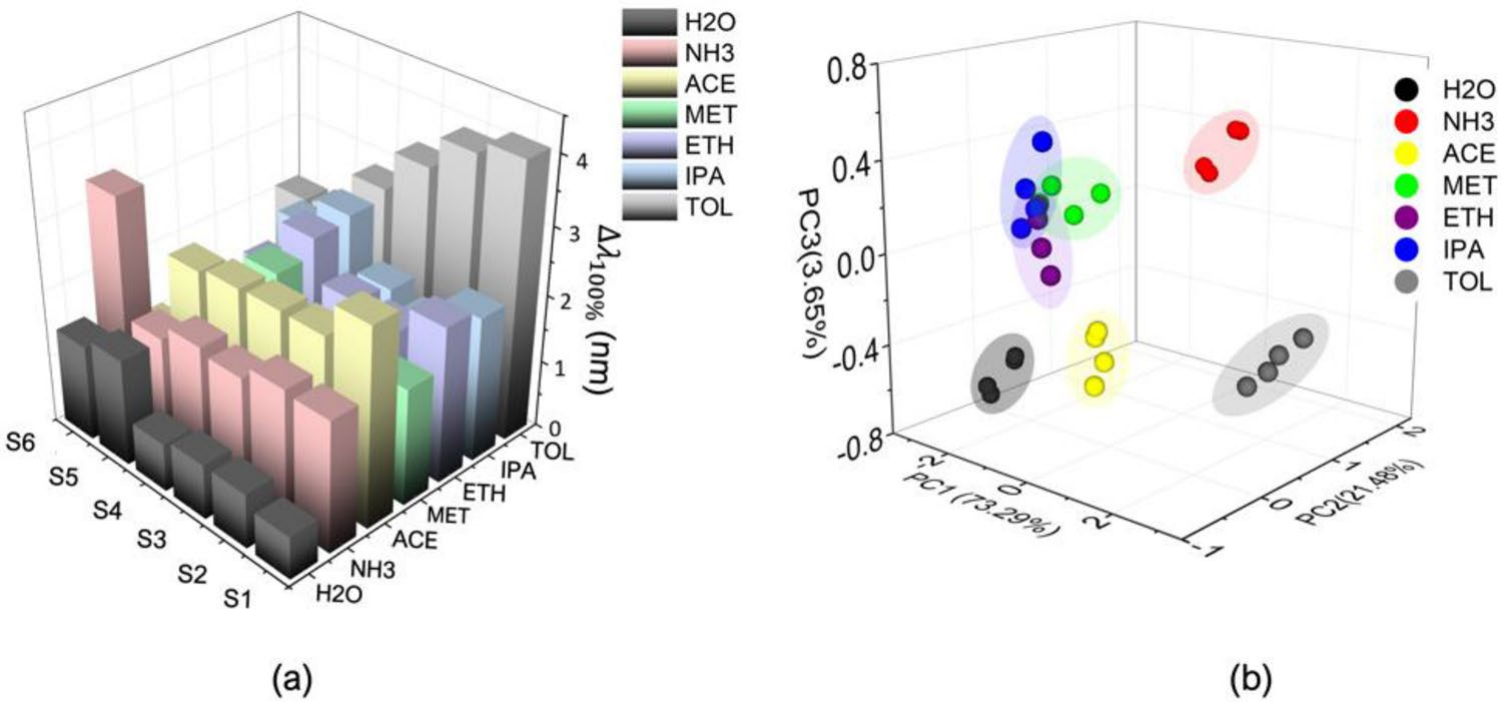
(**a**) 3D bar plot demonstrating the mean values ${\Delta \lambda }_{100\%}$ of six sensors (S1: no GO, S2: 1 mg/ mL, S3: 2 mg/ mL, S4: 3 mg/ mL, S5: 4 mg/ mL, and S6: 5 mg/ mL) when measuring 6 VOCs and DI water at 5% concentrations with 4 record data. (**b**) 3D scatter plot of PCA scores to show classification of the VOC measurements based on the six sensors.

To further analyze the sensors’ selectivity, this section examines the crossed reaction of the proposed pure and hybrid sensors. The time plots of all measurements (as noted in the supporting information S5) are processed using the methods described in Section "Signal acquisition". In addition, the effect from humidity is investigated by replacing the VOC solution with deionized (DI) water. The measurement is then proceeded with similar method at fixed concentration of 5% and four measurement cycles. Following Eqs. (1), (2), and (3), three parameters which ${\Delta \lambda }_{100\%}$, ${\Delta \lambda }_{90\%}$ and ${S}_{c}$ are then obtained for further analysis. The mean values of ${\Delta \lambda }_{100\%}$ from each sensor and each VOC measurement are plotted in the 3D-bar plot Fig. 7a to illustrate distinguishable signal pattern, particularly for NH_3_, acetone, toluene, and DI water. To support this analysis, two approaches of machine learning are employed. First, unsupervised principal component analysis (PCA)^32^ is used to verify cluster discrimination between the VOCs, ensuring that they can be differentiated. This allows for the use of decision tree algorithm^33^ (supervised machine learning) for data prediction.

A 28 × 18 matrix is constructed using the three extracted parameters from each six sensors and six VOC. The PCA function in MATLAB was utilized to generate PCA scores and clusters (see Fig. 7b). The results indicate clear separation among all VOCs and DI water using three principal components (PC1: 73.29%, PC2: 21.48%, PC3: 3.65%). It is worth noting here that the humidity interference may affect the sensor performance, this however can be neglected by the proposed sensor array platform.

Based on the clear separation of VOC clusters, a decision tree algorithm is employed to classify the VOCs based on the sensor metrics. The algorithm is implemented in Python using scikit-learn^34^. As presented in the supporting information S8, the final decision tree relies on 5 metrics of 4 sensors to accurately classify VOCs. The decision tree utilized a 70% training set and a 30% test set with default parameter (Gini impurity criterion) and a depth of 5. The decision tree achieves a 100% accuracy, demonstrating that the developed system can be used for VOC detection.

## Conclusion

In this paper, the hybrid GMR sensing platform by integrating two sensing materials, TaO_2_ and GO, is proposed to enhance the VOC detection. The sensors are formed by spin coating the colloidal GO on the nanocolumnar TaO_2_ GMR sensor. By varying GO concentration, GO is coated on the sensor with different percentage of coverage. This introduces the combination of two sensing mechanisms with and without the assistance of addition functional groups on the sensor. In VOC measurement, the pure TaO_2_-GMR sensor indicates low selectivity, however the signal is directly proportional to the VOC physical properties (molecular weight, vapor pressure, etc.). The hybrid sensors on the other hand become higher selective to methanol when ~ 70% of GO is covered on TaO_2_ waveguide film. At the sensor (3 mg/mL GO coating), the FTIR measurement reports the strong absorbance of epoxy group. The DFT calculations of methanol adsorption on the GO also confirm the close adsorption distance to the epoxy group. For the sensor coated with 5 mg/mL of GO, the sensing mechanisms mainly rely on GO due to 100% GO coverage. The sensor is highly responsive to the NH_3_. Two major functional groups (hydroxyl and carboxyl group) with strong signal are measured by FTIR, while the epoxy group is weakened in comparison to the hybrid sensor of 3 mg/mL GO. The DFT calculations guarantee the NH_3_ adsorption with closest distance to the hydroxyl group. By combining the extracted resonance shift from the proposed sensors, the VOC signals with distinguishable fingerprint is observed. The data classification using principal component analysis (PCA) method indicates the clear data separation. That allows the use of unsupervised machine learning for data prediction. With a decision tree method, the results show 100% accuracy based on 24 data (70% training set and a 30% test set). However, it is important to note the small number of data in this study may have affected the results. Further validation using a larger dataset is recommended to confirm the system accuracy.

### Supplementary Information

Supplementary Information.

## Data Availability

All data generated or analysed during this study are included in this published article [and its supplementary information files].
