# Author Correction: Demonstration of cross reaction in hybrid graphene oxide/tantalum dioxide guided mode resonance sensor for selective volatile organic compound

**DOI:** 10.1038/s41598-023-40864-5

**Published:** 2023-08-22

**Authors:** Khwanchai Tantiwanichapan, Romuald Jolivot, Apichai Jomphoak, Nantarat Srisuai, Chanunthorn Chananonnawathorn, Tossaporn Lertvanithpol, Mati Horprathum, Sakoolkan Boonruang

**Affiliations:** 1 Spectroscopic and Sensing Devices Research Group (SSDRG), Opto-Electrochemical Sensing Research Team (OEC), National Electronics and Computer Technology Center (NECTEC), Pathum Thani, 12120 Thailand; 2 School of Engineering, BU-CROCCS, Bangkok University, Pathum Thani, 12120 Thailand

Correction to: *Scientific Reports* 10.1038/s41598-023-37795-6, published online 04 July 2023

The original PDF version of this Article contained a repeated error where, “Density Functional Theory (DFT)” was incorrectly given as “distribution function theory (DFT)” in the Abstract and Introduction sections.

The original Article has been corrected.

