# Supplementary material for: Demonstration of cross reaction in hybrid graphene oxide/tantalum dioxide guided mode resonance sensor for selective volatile organic compound
Source: Sci Rep. 2023 Jul 4;13:10799. doi: 10.1038/s41598-023-37795-6 (PMC10319844; doi:10.1038/s41598-023-37795-6)
Supplement: Supplementary file 1 — Supplementary Information. [file 41598_2023_37795_MOESM1_ESM.docx]

Demonstration of cross reaction in hybrid graphene oxide/tantalum dioxide guided mode resonance sensor for selective volatile organic compound: supplemental document

S1. GMR fabrication

1. Master mold preparation

The grating master mold was prepared by laser interference lithography (LIL) method. Here, two-beam interferometer set up with HeCd laser (λ = 442 nm) was utilized. The interference angle was set to obtain the desired grating period of 350 nm. The pattern was then recorded on a positive photoresist film (Shipley Microposit S1805) spin-coated on a glass slide at 3000 rpm for 60 s and then soft-baked at 90 °C for 60 s. The interference pattern was exposed on the film with an exposure dose of 100 mJ/cm2 and developed using a Microposit MF- 26A developer. The developed grating was finally hard baked at 90 °C for 60 s.

1. PDMS mold preparation

The pressing mold was casted using a polydimethylsiloxane (PDMS) elastomer (Sylgard 184 silicone elastomer from Dow Corning). The PDMS mixture of the prepolymer and curing agent with a volume ratio of 10:1 was first prepared. The mold-casting protocol was adopted in a vacuum desiccator to ensure that air bubbles were completely removed. Then, it was cured in an oven at 90 °C for 5 hours.

1. Nanoimprint process

Spin-on-glass (SOG) with part number 400F from Filmtronics was used for thermal-curing nanoimprint resist. The SOG film was first spin-coated on a glass slide at 4000 rpm for 90 s. The PDMS grating replica are later pressed on the SOG film at an optimum force (0.176 kg/cm^2^) on a hotplate at 150 °C for 15 minutes.

1. Film deposition by sputtering

The tantalum (Ta: 99.995%) target with 2-inch diameter was sputtered by argon (Ar) atoms while flowing oxygen (which behaves as a reactive gas) into a sputtering chamber to generate an oxide film. The flow rates of Ar and O_2_ were adjusted using a mass flow controller at 15 sccm and 7 sccm, respectively.

S2. Signal processing algorithm


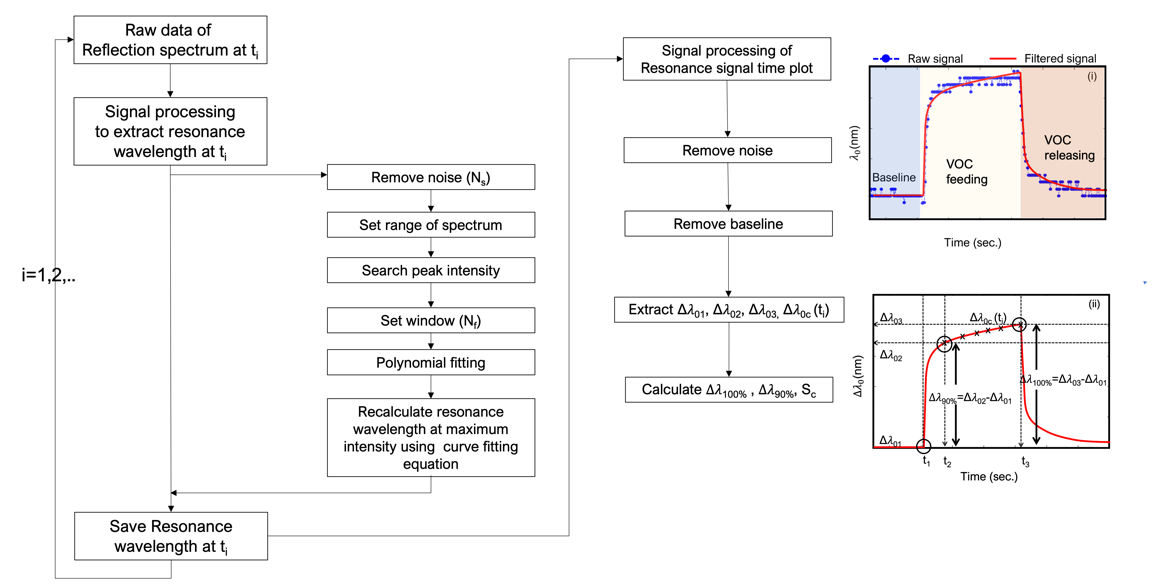


Fig. S2. Flow chart of data processing algorithm.

S3. Sensor’s morphology


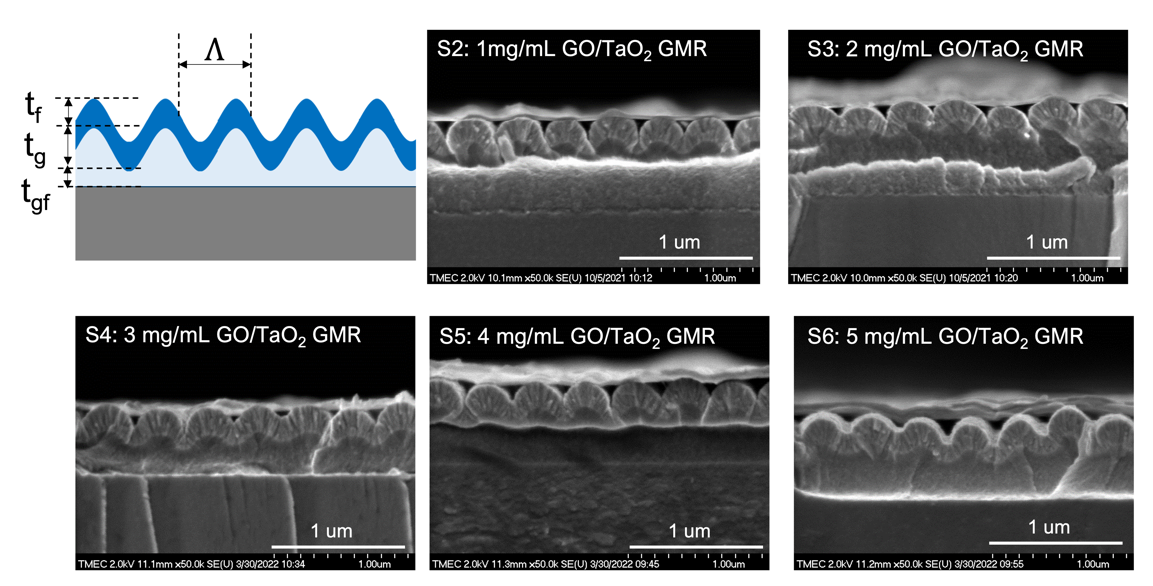


Fig. S3. FE-SEM images (crossed section) of the fabricated GO/TaO_2_ GMR sensors

Table S3. Dimensions of the fabricated GO/TaO2 GMR sensors

| Sensor# | $\Lambda$ (nm) | t_f_ (nm) | t_gf_ (nm) | t_g_ (nm) |
| --- | --- | --- | --- | --- |
| S2 (GO 1mg/mL) | 357±24 | 166±6 | 370±33 | 81±3 |
| S3 (GO 2mg/mL) | 349±14 | 159±6 | 430±13 | 74±15 |
| S4 (GO 3mg/mL) | 364±16 | 209±9 | 214±18 | 79±9 |
| S5 (GO 4mg/mL) | 362±13 | 191±7 | 302±16 | 81±8 |
| S6 (GO 5mg/mL) | 358±10 | 190±13 | 309±28 | 83±12 |

S4. Resonance spectrum


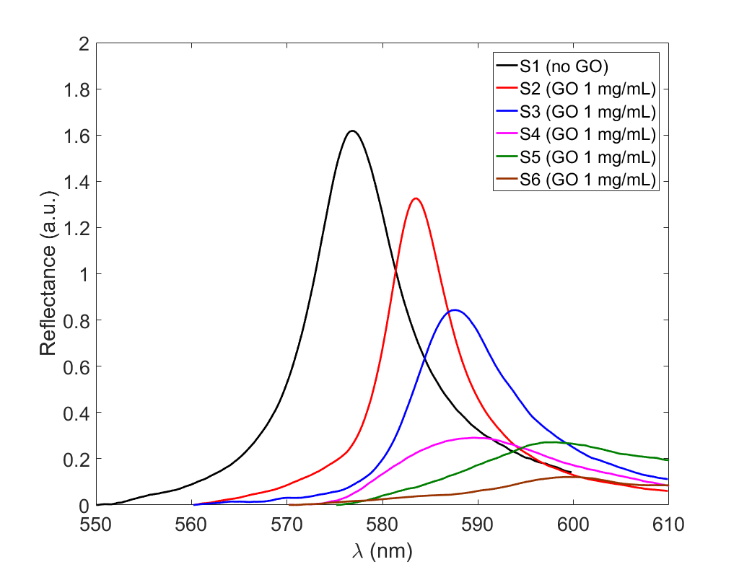


Fig. S4. Comparison of the measured resonance spectrum of the fabricated sensors (TaO_2_-GMR without and with GO coated at 1, 2, 3, 4, and 5 mg/mL in air at room temperature.

S5. VOC selectivity’s measurements


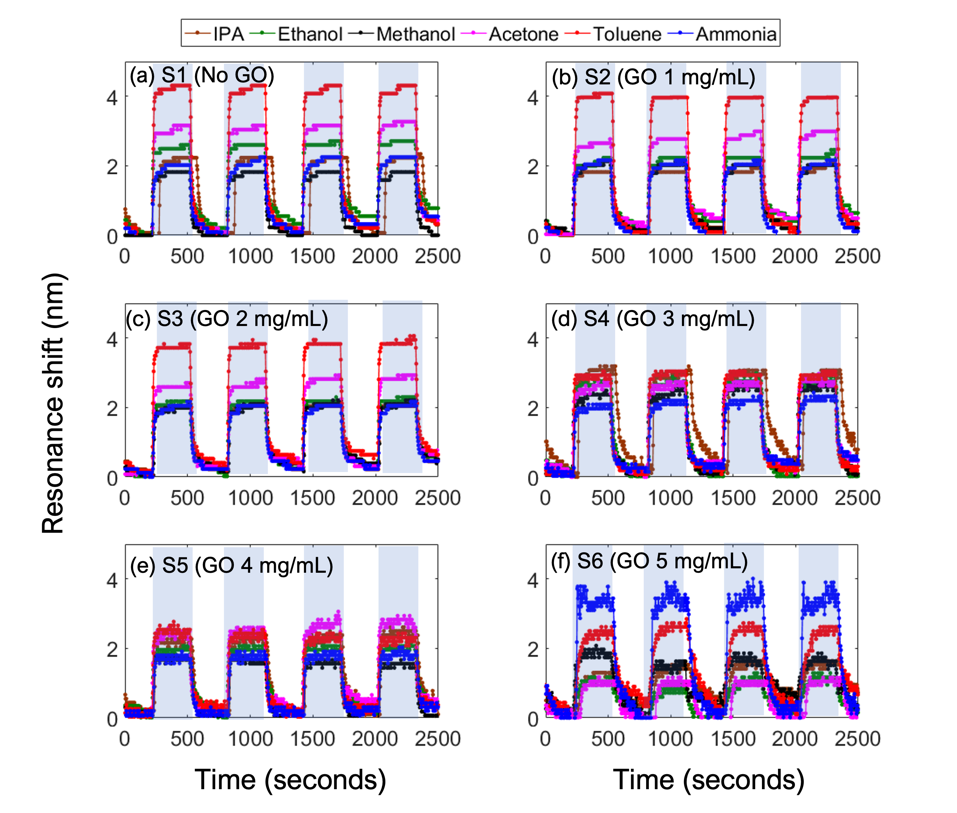


Fig. S5. Compared resonance time plots of of six sensors under the individual measurement of six VOC at fixed concentration of 5% and four measurement cycles (a) S1:no GO (b) S2: GO 1 mg/mL (c) S3: GO 2 mg/mL (d) S4: GO 3 mg/mL (e) S5: GO 4 mg/mL (f) S6: GO 5 mg/mL.

S6. VOC sensitivity’s measurements


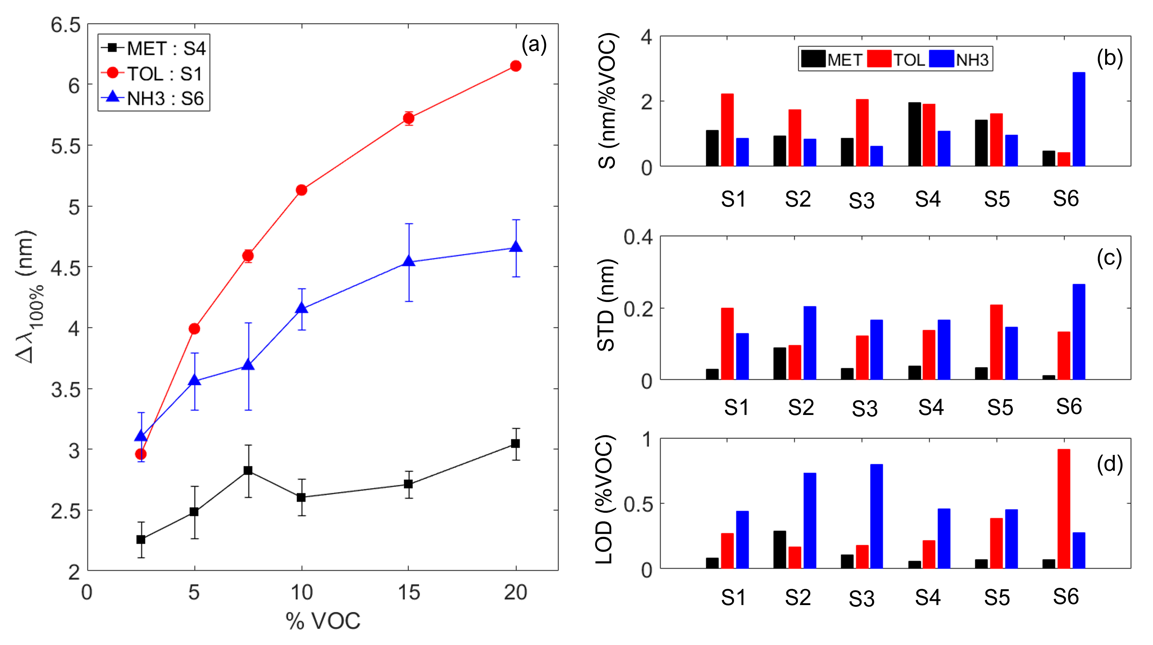


Fig. S6. (a) Comparison plot demonstrating resonance shift versus VOC concentration of S4: GO 3 mg/mL when testing with methanol, S1: no when testing with toluene, S6: GO 5 mg/mL when testing with ammonia (b)-(d) bar plots demonstrating the sensitivity (S), the standard division (STD), and the limit of detection (LOD) of 6 sensors when testing with methanol, toluene, and ammonia. Noted that sensitivity is calculated using the data in linear range (%VOC = 2.5-7.5 %).

S7. Physical properties of VOC

The graph in Fig. S7 shows the trend of the resonance shift in comparison to the molecular weight of the measured VOC. As demonstrated, the resonance signal of the pure TaO_2_-GMR sensor is shifted with the degree directly proportional to the molecular weight of the measured VOC. It is worth noting here that although isopropanol alcohol and acetone have similar molecular weight, acetone has much higher vapor pressure which justifies the higher resonance shift in acetone. Nevertheless, the response of the other hybrid sensor (S4 and S6) does not significantly follow the physical properties of the measured VOC.


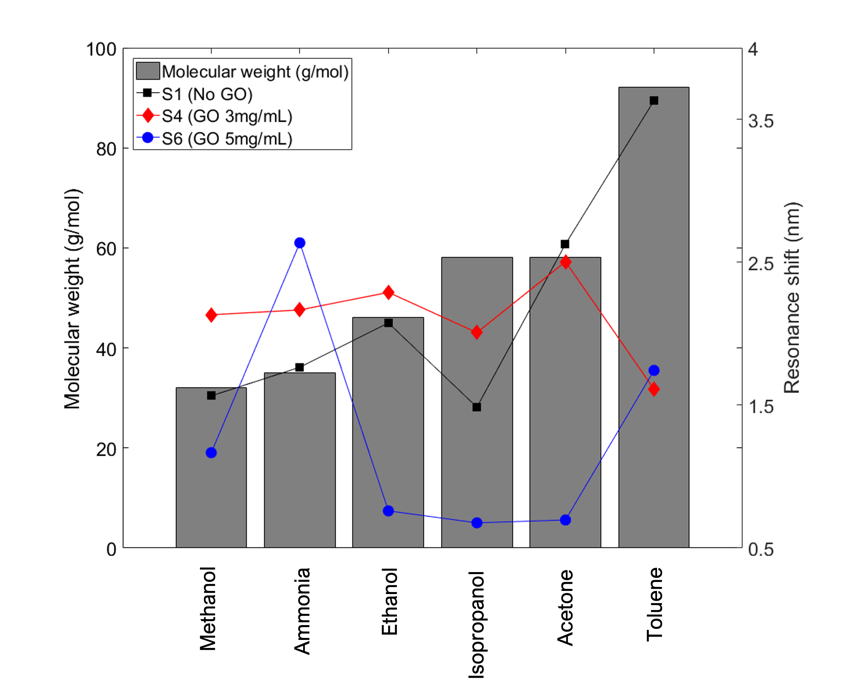


Fig. S7. Comparison plot between VOC molecular weight and measured resonance shift of S1 (No GO), S4 (GO 3mg/mL), and S6 (GO 5 mg/mL).

S8. Decision tree algorithm


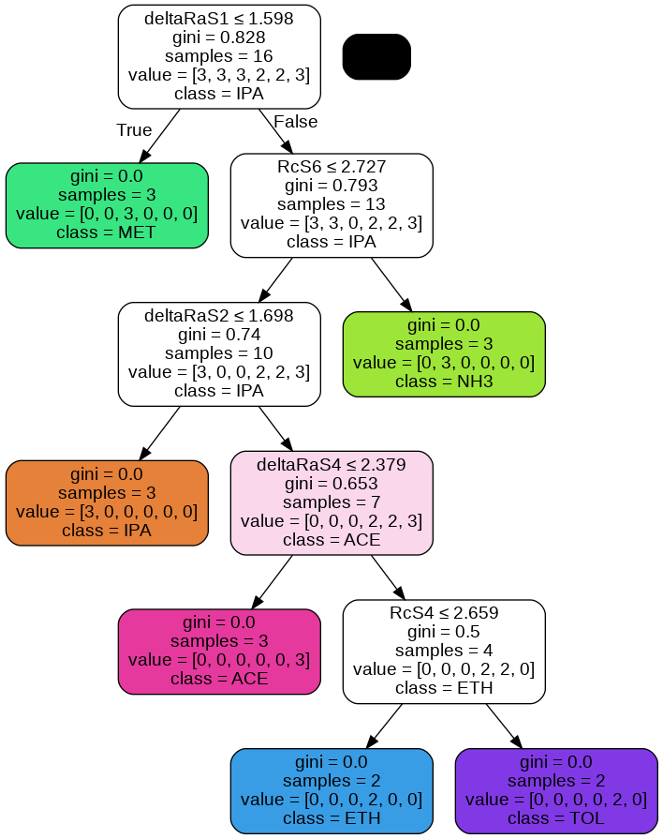


Fig. S8 Decision tree of depth 5, where deltaRaSi and RcSi (i=1,2,...,6) refer to ${\Delta\lambda}_{100\%}$ and $S_{c}$ of sensor S1 to S6, respectively.
